# Defining the Role of Monocytes in Sjögren’s Syndrome

**DOI:** 10.3390/ijms232112765

**Published:** 2022-10-23

**Authors:** Jose Miguel Sequí-Sabater, Lorenzo Beretta

**Affiliations:** 1 Rheumatology Department, Reina Sofía University Hospital, Menéndez Pidal Ave., 14005 Córdoba, Spain; 2 Maimonides Institute for Research in Biomedicine of Córdoba (IMIBIC), University of Córdoba, Menéndez Pidal Ave., 14005 Córdoba, Spain; 3 Referral Center for Systemic Autoimmune Diseases, Fondazione IRCCS Ca’ Granda, Ospedale Maggiore Policlinico di Milano, Francesco Sforza St. 35, 20122 Milan, Italy

**Keywords:** Sjogren’s syndrome, monocytes, inflammation, epigenetics, proteomics, RNA sequencing

## Abstract

Sjögren’s syndrome is one of the most prevalent autoimmune diseases after rheumatoid arthritis, with a preference for middle age, and is characterised by exocrine glandular involvement leading to xerostomia and xerophthalmia. It can have systemic implications with vascular, neurological, renal, and pulmonary involvement, and in some cases, it may evolve to non-Hodgkin’s lymphoma. For a long time, B- and T-lymphocytes have been the focus of research and have been considered key players in Sjögren’s syndrome pathogenesis and evolution. With the development of new technologies, including omics, more insights have been found on the different signalling pathways that lead to inflammation and activation of the immune system. New evidence indicates that a third actor linking innate and adaptive immunity plays a leading role in the Sjögren’s syndrome play: the monocyte. This review summarises the recent insights from transcriptomic, proteomic, and epigenetic studies that help us to understand more about the Sjögren’s syndrome pathophysiology and redefine the involvement of monocytes in this disease.

## 1. Introduction

Sjögren’s syndrome (SS) is a systemic autoimmune disease of unknown origin mostly observed in middle-aged or elderly female adults [1] with a peak incidence between 50 and 60 years [2]. SS [3] can occur independently of other pathologic conditions (primary SS, pSS) or in the context of other autoimmune diseases, mostly rheumatoid arthritis, systemic lupus erythematosus, or systemic sclerosis (secondary SS) [4].

SS is mainly characterised by involvement of exocrine glands, leading to decreased tear production and xeropthalmia (ocular dryness) or decreased salivary flow production and xerostomia (oral dryness) [5]. Fatigue and joint pain are common complaints referred by SS patients and, in most severe cases, internal organs or apparats can be affected as well. SS can indeed involve the kidneys (tubulointerstitial nephropathy and glomerulonephritis), the lungs (interstitial pneumopathy), the neurological system (central or peripheral), and the vascular (vasculitis phenomena) or the haematological system, also increasing the risk of lymphoma [6].

Similarly to other systemic autoimmune diseases, a serum autoantibody positivity and signs of immune system activation and deregulation can be found in most SS patients [7]. An antinuclear antibody (ANA) positivity can be observed in >80% of cases and extractable nuclear antigen (ENA) antibodies in SS patients directed against Ro/SSA and La/SSB antigens can be observed in 33–74% and 23–52% of cases, respectively [8]. Additionally, a serum positivity for the rheumatoid factor (RF) can be observed in 36–74% of patients who also may have hypergammaglobulinemia and indirect signs of B-cell activation, especially in conjunction with anti-Ro/SSA and/or anti-La/SSB positivity [8].

SS is an orphan disease, which is a disease that affects only a very small number of individuals or is neglected by physicians and pharmaceutical companies [9,10], and currently, there is no targeted therapy available to globally tackle this condition. Almost all existing therapies aim at resolving or relieving specific symptoms or specific organic complications [4]. 

The main cell involved in this disease is the B-lymphocyte, which is supposed to have an altered function, being hyper-stimulated by factors/proteins such as the B-cell activating factor (BAFF/BLyS) [11]. The prolonged B-cell activation and proliferation is the key driver of exocrine gland involvement and may result in the appearance of lymphoma, which is one of the most prevalent causes of mortality in SS. As the immune system is composed of several subtly interlaced compartments, more and more studies are shedding light on the role of T-lymphocytes as mediators in the perpetuation of this immune response or as key activators of B-cells [12]. 

Monocytes are cells that play a crucial role in innate immunity, serve as a link to the adaptive immune system, and have been implicated in the development of autoimmune diseases, infiltrating target organs, being altered in number and in function, or releasing cytokines and chemokines with pro-inflammatory, proliferatory, and regulatory functions [13]. Monocytes in SS are, thus, likely part of a puzzle that has yet to fit in. Thanks to the evolution of omics (genomics, proteomics, and metabolomics) [14] and epigenetics (DNA methylation, micro-RNA, and histone modification) [15,16], concepts about the role of different immune actors—including monocytes—in SS are actively updated and revised. The purpose of this review is to investigate the role of monocytes and their different subpopulations in the onset and development of SS, as well as to highlight future therapeutic targets.

## 2. Monocytes

Monocytes are cells that are central both to the innate and to the adaptive immune system and participate in homeostasis and inflammatory responses [17]. Monocytes develop in the bone marrow and, from there, move to the circulatory system to exert their function [18]. They can originate both from monocyte-granulocytic and dendritic-monocytic progenitor cells and, thus, a variant plasticity is attributed to this cell. In relation to the different markers expressed on their surface, we can distinguish different subfamilies of monocytes, each characterised by peculiar functions [19]. In detail, the main subfamilies of monocytes are: 

### 2.1. Classical (CD14^++^, CD16^−^)

Classical monocytes are by far the most represented population of monocytes in blood (about 80–90%) and are considered inflammatory [20]. In humans, classical monocytes highly express CD14 and lack CD16; they also express the C-C chemokine receptor type 2 (CCR2 or CD192), a molecule necessary to exit from bone marrow to the circulation. Other surface markers have been described to foster a more precise definition and isolation of classical monocytes [21], including the increased expression of the scavenger receptor CD36 [22] and the reduced expression of the CD11c [23]. Within this subpopulation, different subtypes can be further distinguished in relation to other surface markers and function. Classical monocytes expressing the CD103 have tumour-antigen-presenting functions [24]. MHCII+, SCA-1+, and CXC3R1- monocytes can produce prostaglandin E2 (PGE_2_) and interleukin 10 (IL-10) in the case of infection [25]. Dendritic cells (DCs) from MHCII+ and CD209a+ monocytes [26] also represent a subpopulation of monocytes found in small numbers (5%) and described in inflamed peripheral tissues, but not in lymphoid tissue, and is partially CCR2-dependent. These monocytes are differentiated via interferon gamma (IFN-γ), which, in this case, is secreted by natural-killer (NK) lymphocytes in response to inflammation. They play a co-stimulatory role with CD8+ T-lymphocytes. Their function is superimposable to that of type 2 DCs, which explains why, in the absence of these, the latter, monocytic-DC determines its DC function and can even present the antigen to lymphocytes, although with less efficiency. Segregated-nucleus-containing atypical monocytes (SatMS) CD115+ monocytes have been shown to appear de novo in experimental models of fibrosis after exposure to bleomycin [27], being critical for fibrosis development.

### 2.2. Nonclassical (CD14^−^, CD16^++^)

Nonclassical monocytes express high levels of the adhesion-related-receptor C-CX-C motif chemokine receptor 1 (CX3CR1) and actively patrol the vasculature both at steady state and during inflammation to remove debris [28] and produce high levels of anti-inflammatory cytokines and pro-wound-healing factors [29]. They exert patrolling functions under either NOTCH2 and/or toll-like receptor 7 (TLR-7) stimulation in inflammatory conditions [30]; patrolling is, in part, dependent on the expression of the lymphocyte-function-associated antigen 1 (LFA1) on monocytes [31].

Within this population of monocytes, several subsets have also been described [32], including a CD61+ and 6-Sulfo LaNAc (Slan)-positive subset associated with coronary artery disease severity or a CD9+ subset involved in platelet adhesion. A slan- subtype has also been described, although it does not seem to be functionally different from the slan+ subset at the transcriptional level [32]. Additionally, a subset of monocytes expressing the angiogenic tyrosine-protein kinase receptor Tie-2 has been described to promote angiogenesis in tumours, possibly playing a role in human cancer progression [33,34].

### 2.3. Intermediate (CD14^+^, CD16^+^)

A third major subtype that is halfway between classical and nonclassical monocytes has consistently been described. This subset is part of a continuum as classical monocytes can re-convert into nonclassical monocytes, with intermediate monocytes representing a transition point: a classical monocyte in 1–2 days can become an intermediate after a stimulus and subsequently transform into a nonclassical monocyte [35]. Intermediate monocytes highly express antigen-presentation molecules [32] and CCR5 to a higher extent as compared to classical monocytes [36] and are involved in antigen processing and presentation and transendothelial migration.

## 3. Monocyte Activation

After an inflammatory stimulus, a complex system with many factors starts to operate to initiate emergency myelopoiesis [37]. The signals that trigger this process include cytokines and TLRs that directly differentiate macrophages. These signals influence haematopoietic stem cells and multipotent progenitor cells; as a consequence, the number of myelocytes increases, which are released from the bone marrow into the peripheral blood via CCR2 signalling. From this moment on, inflammatory markers increase (nitric oxide, IFN pathway, tumour necrosis factor (TNF), and Janus kinase-signal transducer and activation of transcription (JAK-STAT) pathway), leading to monocyte differentiation under the drive of CD8-lymphocyte stimuli (IFN-γ) [37].

During inflammation, classical monocytes can differentiate into CD11a+ MHCII+ monocytes (macrophages) or monocyte-derived dendritic cells (MoDCs), which can acquire protective or pathological functions [18]. These monocytes depend, among other molecules, on the interferon receptor factor 5 (IRF-5) signalling to differentiate into Podophyllotoxin and Rutin Modulate M1-positive (iNOS+)-producing cells; if this does not happen, they can further differentiate into M2-type CD206+ macrophages with anti-inflammatory properties [38]. Therefore, IRF5 promotes the emergence of M1 (inflammatory) and represses the M2 (anti-inflammatory) lineage, as a result of which pathogenic monocytes or macrophages may develop. In the presence of chronic or sustained inflammation, monocytes may differentiate into macrophages with haemophagocytic activity, leading to the development of a macrophage activation syndrome (MAS) [39].

## 4. Interferon Signature in Sjögren’s Disease

The increased expression of type-1 IFN-regulated genes is commonly referred to as the “interferon signature” [40]. The type-1 IFN pathway mainly stimulates the IFN α/β Receptor (IFNAR) that, downstream, activates the JAK-STAT pathway (Jak1 and Tyk 2), and phosphorylates STAT 1 and 2 together with the mitogen-activated protein (MAP) kinase pathway (Erk 1 and Erk 2) [34].

IFN-1 inhibits viral replication, activates NK lymphocytes and dendritic cells, and maintains the antibody response by B cells [41].

Each type of monocyte reacts differently to IFN stimulation; indeed, after IFN stimulation, the expression of STAT1, as well as the surface expression of CD169 and CD64, is higher in classical monocytes as compared to the other subsets [42]. While nonclassical monocytes that express CD169 on their surface show less affinity/sensitivity for the interferon signature [34], they also show a decrease in STAT1, which results in a decrease in IFNAR1 and its related proteomics [43]. Part of its anti-inflammatory function is due to its role as a cell debris scavenger, thus preventing a continued TLR-mediated IFN-1 stimulation [44]. 

The CCR2-CCL2 molecule mediates the recruitment of classical monocytes following inflammatory stimuli [45], which, together, with the production of the IFN signature, creates the ideal environment for the development of autoimmune diseases. For instance, in systemic lupus erythematosus (SLE), it was found that monocytes were hypersensitive to the IFN receptor signal, thus increasing the number of classical monocytes and the classical vs. nonclassical monocytes ratio [34]. These alterations create an inflammatory environment that is uncontrolled by nonclassical patrolling functions and that further promotes autoimmunity.

In pSS, there is evidence of an upregulation of type-1 IFN genes in circulating monocytes; this finding is consistent with the description of IFN activation in salivary gland monocytes and peripheral blood mononucleated cells (PBMCs) [41]. Additionally, type-1 IFN was found to induce the expression of BAFF expression in monocytes and salivary gland epithelial cells [46]. In this same study, an attempt was made to find a link between BAFF and IFN-1 expression. It was found that after IFN-1 stimulation, monocytes (CD14+) increased BAFF mRNA levels and, once the stimulus was blocked, BAFF levels decreased, although BAFF expression could not be correlated with IFN-1 levels nor serum BAFF be correlated with BAFF mRNA in monocytes or PBMC.

Plasmacytoid dendritic cells (pDCs) are the main producers of type-1 IFN [41]; in pSS, their blood levels were found to be low, most likely as a consequence of margination and migration to exocrine glands [47]. pDCs react to exogenous (viral DNA/RNA) or endogenous stimuli, such as immunocomplexes of nuclear antigens and antibodies (a pSS hallmark) that bind to FcγRIIa, causing its internalisation and intracellular binding to TLR7 and TLR9 [48]. This pathway of IFN-1 production due to immunocomplex binding may provide an explanation for the association between type-1 IFN values with high autoantibodies, IgG production, and low complement in pSS.

## 5. Monocytes in Sjögren’s Syndrome

Monocytes in SS may be studied in different compartments and by different methods; we can either study their presence by histological (salivary gland biopsy and eye biopsy) or blood (peripheral blood, PBMC) samples [49].

**Figure 1:**
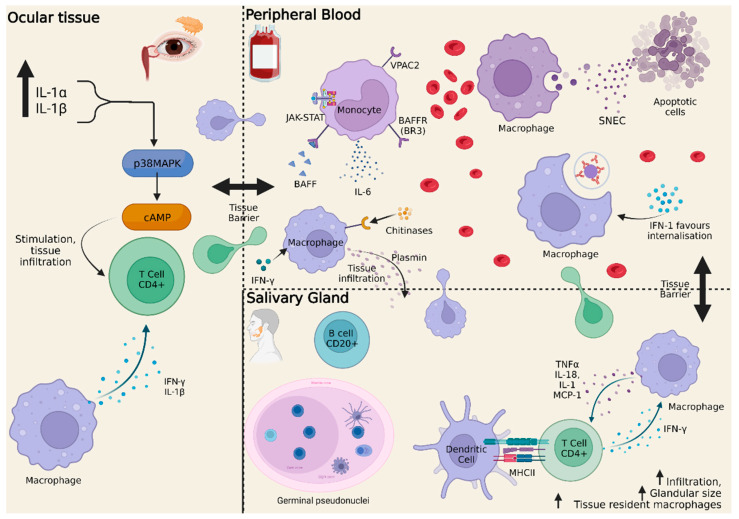
Monocyte and macrophage implication in Sjögren’s Syndrome. This figure shows how the monocyte/macrophage infiltrates peripheral tissues, namely ocular and salivary, interacting with B and T cells, and sustaining inflammation, disease progression, and organ disease damage. IL-1α, Interleukin-1α; IL-1β; IL-18; IL-6; p38 MAPK, p38 MAP-Kinase; cAMP, cyclic Adenosine monophosphate; IFN-γ, Interferon-γ; IFN-1; TNFα, Tumour necrosis factor α; MCP-1, Monocyte chemotactic protein 1; SNEC, secondary necrotic material; MHCII, Major histocompatibility complex type II; JAK, Janus-kinase; BAFF, B cell activating factor belonging to TNF family; VPAC, Vasoactive intestinal peptide receptor. Created with www.BioRender.com. Accessed on 17 October 2022.

In salivary gland biopsies (Figure 1), an inflammatory infiltrate initially composed of CD4+ T-lymphocytes has been described, which, as the disease progresses, gives way to B-cells infiltrates that eventually lead to the formation of germinal pseudonuclei [50]. Within these nuclei, macrophages/monocytes as well as dendritic cells, in addition to lymphocytes, can be found. Their infiltration into the tissue is variable depending on the stage of the disease or the type of cell labelling. Nonetheless, the degree of infiltration was found to correlate positively with the degree of infiltration and involvement of the gland [49]. Macrophages arguably constitute a nexus between innate and adaptive immunity, connecting the different players in glandular involvement. Macrophages secrete a variety of inflammatory cytokines and mechanisms (including IL-18, IL-1, and MCP-1) and proteases in the process of monocyte–macrophage differentiation, activation of lymphocytes, or TLRs of innate immunity [49]. On the other hand, TCD4+ lymphocytes are stimulated by local dendritic cells via MHCII and activate tissue-resident macrophages through the production of IFN-γ and other cytokines [51]. This process increases the inflammatory infiltrate and eventually increases the size of salivary glands and leads to trophic changes into the affected tissues.

In the case of keratoconjunctivitis sicca (ocular involvement), elevated IL-1α and IL-1β can be seen in the conjunctival tissue of pSS, causing pathological keratinisation of the eye [52]. This interleukin activates the MAPKinase pathway (p38 MAPK), which leads to the transcription of cyclic AMP (inflammatory pattern), maintaining the activation of resident cells based on CD4+ infiltration.

The balance between classical and nonclassical monocytes is fundamental to regulate inflammation. As described above, nonclassical monocytes have the function of removing apoptotic cell debris, thus preventing antigen presentation and immune system activation [28,53]. In extreme conditions, a prolonged presentation of (auto) antigens may sustain autoimmunity. This is the case, for instance, of SLE where the clearance of apoptotic debris, including alarmins, via DNAse 1 is defective [54]. The accumulation of these molecules, called secondary necrotic material (SNEC), is phagocytosed by macrophages and neutrophils, which, upon activation, secrete cytokines and stimulate intracellular DNA sensors and RNA-associated nucleoproteins [55]. Both in pSS and in SLE, macrophages are overstimulated by a favourable serum environment that leads to a higher phagocytosis than under normal conditions [54]. In pSS, a significant correlation has indeed been found between defective DNAse and SNEC degradation, and disease activity was found to inversely correlate to DNAase levels [54]. As part of a complex system, autoantibodies also play a crucial role in preventing the degradation of SNEC compounds against endonucleases, creating a kind of protective film that keeps the antigen intact until phagocytosis [56]. Additionally, type-1 interferon-related pathways favour the internalisation of immunocomplexes, facilitating the appearance of nuclear self-antigens [57].

Other factors produced by macrophages/monocytes in local tissues may also play an essential role in the pathogenesis of SS, including chitinases, plasmin, and cathepsins. Aberrant expression of human chitinase genes, including the chitinase-3-type-1 (CHI3L1/YKL-40) and the chitinase 1 (chitotyrosidase/CHIT1) gene, has been demonstrated in histological salivary gland biopsy specimens with increased expression corresponding to a more severe disease in pSS [58]. This process is involved in monocyte adhesion as well as differentiation into macrophages, and may be a direct activator of infiltration and disease development. Plasmin, a serum protease product of plasminogen activators, is actively produced by macrophages after stimulation by IFNγ; it can lead to fibrinolysis and is capable of activating metalloproteases, which weaken the matrix of the affected tissue and facilitate lymphocyte invasion, thereby establishing tissue damage [59]. Cathepsins, also proteolytic in nature, are endosomal/lysosomal peptidases, which are activated at low pH [60]. Increased cathepsin S, as well as cathepsin H, has been found in the lacrimal glands together with CD68+ macrophages [61].

Another molecule of interest in pSS pathogenesis is the vasoactive intestinal peptide (VIP); this molecule generates immunomodulation via its receptors VPAC1 and VPAC2 in macrophages, monocytes, and T-lymphocytes [62]. pSS monocytes express increased VPAC2, whose presence is associated with impaired phagocytosis [63]. In a study with NOD mice with Sjögren-like manifestations, VIP administration was tested, restoring salivary secretion and reducing markers of autoimmunity [64]. At the cellular level, a change in the proportion of predominantly M1 macrophages (pro-inflammatory) to M2 (noninflammatory) was also observed. Moreover, classical monocytes located in salivary glands after phagocytosis show increased TNF-α levels, that is, the opposite of what is usually observed in normal conditions where monocytes/macrophages after phagocytosis of apoptotic cells secrete anti-inflammatory cytokines, with low levels of TNF-α and high levels of IL-10.

The autoimmune regulator (AIRE) is a gene whose gene transcription factor is mainly found in the thymus and whose protein coordinates the expression and presentation of tissue-specific self-antigens, thus constituting an essential role for the depletion of autoreactive lymphocytes in the thymus [65]. It has been observed that AIRE knock-out mice (KO) can develop an autoimmune disease mediated by CD4+ lymphocytes that can affect multiple organs, including exocrine glands, which is superimposable to pSS. Keratoconjunctivitis sicca, xerophthalmia, and peripheral neuropathy, among others, have been reproduced in these mice. In the glandular infiltrates of these mice, a heterogeneous population of immune cells has been observed, comprising CD4+, CD8+ and B-lymphocytes, DCs, as well as macrophage/monocytes. Macrophages/monocytes of these infiltrates highly express pro-inflammatory cytokines such as IL-1β and IFN-γ in the ocular tissue.

Monocytes tightly interact with B-lymphocytes, a major player in pSS pathogenesis [66]. Several studies have shown that the BAFF receptor BR3 is over-expressed in peripheral monocytes, especially classical monocytes, which can actively secrete IL-6 after BAFF binding, and this mechanism is blocked by anti-BAFF antibodies [67]. Such BR3 expression may play a role in the expression of immunoglobulins as well as autoantibodies; in fact, exposure of monocytes to anti-IL-6 has been tested, inhibiting the levels of this interleukin. This finding suggests that soluble factors that are produced by BAFF-stimulated monocytes are involved in the production of immunoglobulins by IL-6-activated B-lymphocytes [68]. 

In addition to the IFN pathway, MAPKinases and the JAK-STAT pathway have gained prominence either as key players in the pathogenesis of pSS or as therapeutic targets [69]. The JAK-STAT pathway mediates cytokine responses, including IL6, IL-7, IL-10, IL-12, IL-17, IL-21, and TNFα, all implicated in the pathogenesis of pSS. The JAK-STAT pathway is involved in IFN signalling as STAT tyrosine phosphorylation follows JAKs activation downstream of IFN receptors [70]. A recent study has analysed STAT1, 3, 4, 5, and 6 in peripheral blood, in T- and B-lymphocytes and monocytes in patients from pSS and healthy volunteers [71]. Phosphorylated STAT5 in monocytes as well as in B-lymphocytes strongly correlated with IgG and anti-SSB/La serum levels and, from the clinical point of view, with the presence of purpura but not with other clinical features. Regarding the other STAT molecules, STAT4 was shown to be predisposed to pSS and to affect the type-1 IFN pathway, while STAT1 and 3 can be found overexpressed at the mRNA level in PBMC. 

### 5.1. Transcriptome Findings

In SS, recent transcriptome studies have been performed to investigate potential pathways of interest and to elucidate the pathogenesis of the disease [46]. In RNA, from CD14+ monocytes, statistically significant differences in TNFSF10 (TRAIL) expression were found in patients with pSS vs. healthy controls. The following differentially expressed genes (DEGs) were also highlighted: TMEM176B, TMEM176A, HLA-DRB5, FOS, TXNIP, ARPC1B, GRN, FGL2, SAMHD1, CEBPD, CTSZ, HLA-DQB1, SNX17, TNFSF10, WASF2, ATP5A1, ZFP36L2, and CORO1A. Among them, HLA DRB5 and TNFSF10 play a key role in the pathogenesis of many autoimmune diseases [72,73]. Enrichment/stimulation analysis of these DEGs identified neutrophil activation and IFN-associated pathways. 

More studies [74], comparing the bulk transcriptome in CD14+ genes from SLE and pSS patients, showed that inflammatory as well as IFN pathways were enriched in both diseases. A propensity toward M1 macrophage differentiation appeared to be prominent in pSS.

In pSS, bulk transcriptome analysis identified a significant number of genes aberrantly expressed in monocytes: TRIM22, MX2, MS4A4A, IFI44, IFIT2, STAT2, SAMD9L, STAT1, EPST11, IFI44L, SIGLEC1, TNFSF10, CX3CR1, and ISG15 [75]. Among them, we can appreciate several associated with the IFN pathway with anti-viral and inflammatory properties (STAT, IFIT, TNFSF, among the others). Increased expression of TNFSF10 (TRAIL) in patient monocytes was identified both in scRNA-seq and bulk transcriptomic analyses. As this gene is expressed in many of the identified monocyte subsets, these data reinforce the previously described finding that TNSF10+ may play a key role in the pathogenesis of pSS, as also suggested by other studies [76,77].

Similarly, comparing the transcriptome of CD14+ monocytes from patients with pSS, non-Sjögren’s sicca (nSS), and healthy controls (HC), it was observed that the gene expression of circulating monocytes (especially intermediate and nonclassical monocytes) was highly correlated with ongoing systemic inflammation as result of local damage [78]. Overall, the expression profile of pSS was clearly distinct compared to HC monocytes, but had relatively similar profiles to nSS monocytes. Treatment with serum from pSS patients induced pSS-like transcriptome features in hallmark genes in HC-derived monocytes; these effects were mostly driven by type-1 IFNs.

The literature [41,79] reports similar results describing type-1 IFN genes (IFI44L, IFI44, IFIT3, LY6E, and MX1) overregulated in monocytes with pSS and associated with high disease activity. Other genes are related to the IFN pathway such as IFI27, IFITM1, IFIT4, and IFI44 as well. Sialic acid binding Ig-like Lectin 1 (Siglec-1), which is a biomarker of type-1 IFN activation, was highly expressed in monocytes and could also be correlated with EULAR Sjögren’s syndrome disease activity index (ESSDAI) [80]. The study [75] suggested that both the IFN and virus-infection response pathways are over-regulated in pSS monocytes and play a role in its pathogenesis.

### 5.2. Epigenetic Findings

We refer to epigenetics when there are mitotic modifications that can influence the phenotype without the need to alter the DNA sequence. These are relatively stable changes over time that maintain the cellular identity and arise in response to different internal or external stimuli. DNA methylation, histone modifications, and noncoding RNAs (miRNAs) are commonly studied among the epigenetic processes.

The role of monocyte miRNAs in certain pathological processes or for cell regulation [81] influencing the heterogeneity of monocytes has recently been the focus of different studies. miRNAs are molecules with a regulatory role in gene expression at the post-transcriptional level [82]. Almost half of them are clustered together and can be transcribed independently or simultaneously [83].

Many studies have found an overexpression of miR-146a/b in PBMCs from patients with pSS [84,85], and a regulatory role in the immune response via negative feedback of TLR signalling has been proposed [86]. Therefore, a dysregulation of miR-146 would promote uncontrolled inflammation, leading to the phenomenon of autoimmunity. Other studies identified overexpression of miR-181a in PBMCs in contrast to the findings in salivary tissue, in which it was found to be underexpressed together with miR-16 [87] leaving open the need for further studies to investigate a possible pathogenic factor.

In light of the involvement of miRNAs in pSS, attempts have been made to establish a sort of "miRNA signature" not dissimilar to the IFN signature found elsewhere [88]. In the study by Williams et al. [88], six miRNAs were found to be simultaneously overexpressed in pSS (miR-34b-3p, miR-300, miR-609, miR-877-3p, miR-3162- 3p, and miR-4701-5p).

The involvement of these miRNAs in the transforming growth factor β (TGFβ) pathway was studied, recognising that TGFβ is an underappreciated pathway and could have a relevant role in the pathogenesis of pSS. Indeed, female TFGβ1-R KO mice develop inflammation in salivary glands, and TFGβ2-R2 depletion in dendritic cells resulted in multi-organ inflammation and activation of autoreactive T- and B-lymphocytes as well as in a mismatched polarisation of alternatively activated M2 macrophages. Key factors in this pathway are TGFBR3 and SMAD2, direct targets of miR-609 and miR-877-3p, respectively. Of note, expression levels in pSS monocytes of SMAD2 and 3 are high, although SMAD4 is usually reduced. Regression analyses indicated that there was a significant association between miR-300 and miR-609 on the reduction in SMAD4 expression, thus being its target and modulating its expression. The MAPKinase pathway may be regulated by pSS-related miRNAs. The direct effect of miR-34b-3p on GRB2, P38/MAPK13, and MEKK1/MAP3K1, as well as miR-877-3p on SOS1, NRAS, ERK1/MAPK1, RAC1, and HGK/ MAP4K4, was studied. Likewise, with the JAK-STAT pathway: miR-877-3p regulates STAT6, which codes for the IL-4/IL-13 p transcription factor [88]. No relationship was found with the IL-12 receptor (IL12RB1 and IL12RB2), TYK2, or STAT4, related to IL-12 signalling. Regulation of the TLR/NFκβ pathway was also not found; as communication between the NFκβ and TGFβ pathways is essential to coordinate cellular responses and prevent autoimmunity [89], a deficient functioning mechanism between these pathways can trigger pro-inflammatory factors in pSS.

Histones, partners in chromatin compaction in the nucleus, can undergo modifications that will involve one gene expression or another [90]. The N-terminal tails of histones protrude out of the nucleosome and are subject to a variety of post-translational covalent modifications through acetylation and methylation of the lysine residues in histones H3 and H4, the latter being the most studied modifications [91]. Acetylation leads to relaxation of the chromatin conformation in a way that allows transcription, whereas de-acetylation represses transcription by compacting the chromatin. In a review, Imgenberg-Kreuz et al. reported an association between genetic risk variants with promoter and enhancer marks in B-lymphocytes and monocytes [92]. Hypomethylated sites in pSS were observed to accumulate in enhancer regions of T- and B-lymphocytes, while hypermethylated regions predominantly overlapped with a histone mark indicating activation of gene transcription [92]. Despite this evidence, no studies are currently available to analyse histone marks in pSS cells.

Luo et al. [93] proposed that the IFN signature could be detected at the level of DNA methylation and other pathways involved in pSS pathogenesis. DNA methyltransferase 3A (DNMT3A) and methylcytosine dioxygenase translocation 10-11 (TET) play a basic role in the incorporation and oxidation/removal of methyl groups on cytosines [94]. In the case of the monocyte, DNMT3A and TET are related to differentiation and the inflammatory response, respectively. This is an epigenetic mechanism that could reflect the influence that monocyte-mediated inflammation exerts on pSS. In this study, it was found that circulating monocytes in pSS were predominantly DNA-hypomethylated (299 out of 460 genes, 65%). Hypomethylation in MX1, PARP9, DTX3L, EPSTI1, and IFITM1, which influence the IFN pathway in pSS monocytes, was reported. These findings may support the idea that DNA methylation in IFN-signature-related genes could be a diagnostic tool for pSS in peripheral blood. This hypomethylation trend may be a trigger for dysfunctional activation of its related genes, which could elevate the IFN signature in pSS.

Differentially Methylated Positions (DMPs) corresponding to 12 genes that overlapped in pSS monocytes and salivary gland epithelial cells (SGECs) were also described [93]. These include: PTPRN2, TNK1, WDR8, TSPAN9, VIPR2, OBSCN, KCNT1, ZNF703, NEURL3, LMX1B, LOC146336, and FTSJD2. All these DMPs were related to the cell cycle, senescence and the IL-17 pathway. In the same paper, the methylation status was compared between patients who were single positive (for either anti-Ro/SSA or -La/SSB) and those who were double positive (both antibody specificities). Among single positives, only 54 DMPs related to 27 genes were found, of which 9 showed significant differences in methylation with controls. In contrast, in double positives, 1230 DMPs related to 984 genes were found, and of those, 113 showed differences in methylation in the promoter region. The double positives were related to Ras, Ribosomal, Rap1, and AMP-activated protein kinase (AMPK) signalling pathways, while the single positives only showed the NOTCH pathway. Such NOTCH genes have been described in monocytes from other autoimmune diseases (including rheumatoid arthritis) [95], and their hyperactivity may increase macrophage differentiation and promote the production of pro-inflammatory cytokines [96]. 

Differentially methylated genes mostly expressed between the two subtypes were in the ribosome and AMPK pathway. The AMPK-STAT3 axis plays a pivotal role in regulating monocyte-to-macrophage differentiation through increased AMPK activity [97], and thus, changes in methylation may influence IgG production by affecting monocyte differentiation. Furthermore, the NOTCH pathway (DT3XL) was highlighted as an enriched pathway in patients with elevated IgG levels. 

## 6. Current and Future Treatments

Current existing drugs for Sjögren’s syndrome are not targeted to the treatment of this disease and several compounds developed with other indications have often been used on the basis of personal or anecdotical experience, yet with limited clinical efficacy [98].

Immunosuppressants are widely used in pSS, according to the notion that immunological disturbances can be addressed or, hopefully, reverted by this class of drugs even if their effect is unspecific. For instance, hydroxychloroquine has shown limited utility and no substantial effect in controlled clinical trials [99] despite its potential to interfere with the TLR system and to inhibit type-1 IFN responses. Methotrexate, a potent immune-suppressive drug with multiple effects on both acquired and innate immunity pathways, has been poorly studied in pSS despite its broad use in the rheumatology field. In the few studies conducted in pSS, methotrexate showed disappointing and conflicting results, with beneficial effects on haematological and indirect parameters of immune system activation but not on clinical symptoms [100]. Azathioprine has a long history in the treatment of autoimmune diseases, directly enhancing apoptosis of both memory and naive T cells and secondarily inhibiting B cell activation; in pSS, it is widely used as a steroid-sparing agent, yet in small double-blind randomised clinical trials, it did not show any substantial clinical effect [101]. Cyclosporine, a calcineurin inhibitor, specifically targets T cells and related responses, and has been tested in a small trial in pSS showing some limited benefit only on xerostomia, but not on other clinical parameters [102]. Leflunomide possesses the ability to inhibit the proliferation of B cells and both naive and memory CD4+ T cells; in a small trial in pSS, it ameliorated several biohumoral parameters, including cytokine and histological alterations, and proved effective in about 50% of patients when clinical domains were globally considered [103,104]. Mycophenolate mofetil due to its tolerance and low side-effects, as well as efficacy in some clinical manifestations of pSS, has been suggested as a potential first-line therapy, yet large studies to confirm and well assess its efficacy are lacking [105].

B cell over-activation is one of the hallmarks of pSS and drugs targeted toward this cell subset have long been considered a key target for intervention. Rituximab, a depletive anti-CD20 monoclonal antibody, was first described as beneficial in some series of pSS patients [106,107], but a substantial effect was not confirmed in a larger controlled study [108]. Belimumab, an anti-BAFF/BLyS monoclonal, seems effective to reduce systemic activity, parotid enlargement, lymphadenopathies, articular manifestation, and B cell biomarkers in pSS even if concluding evidence is still lacking [109]. Sequential therapy with rituximab and belimumab has been described in selected pSS complications, including cryoglobulinemia [110] and lymphoma [111]. Other B cell-targeted therapies are currently being studied, such as Ianalumab, a BAFF receptor fully human monoclonal antibody, engineered for direct antibody-dependent cellular cytotoxicity-mediated B-cell depletion that was found to be beneficial on clinical domains in a phase II trial [112].

**Figure 2:**
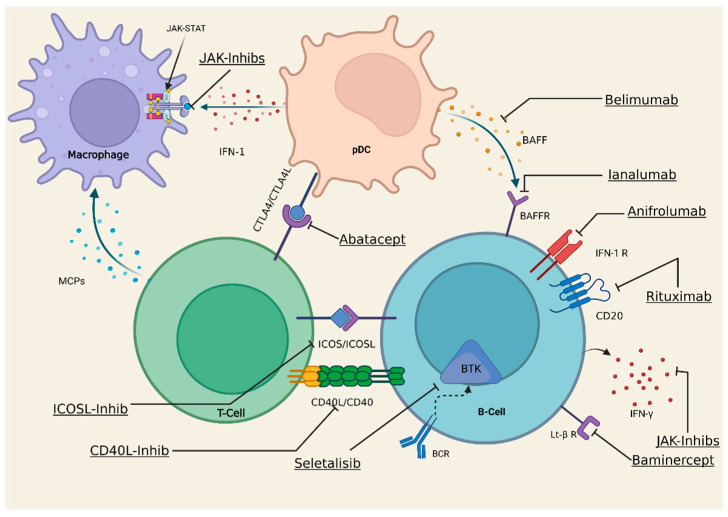
Sjögren’s syndrome targets and future treatments. This figure illustrates some current or potential therapeutic target in relation to immune cells involved in SS pathogenesis. IFN-1, Interferon-1; IFN-1 R, Interferon-1 Receptor; IFN-γ, Interferon-γ; pDC, plasmacytoid dendritic cell; BTK, Bruton-Kinase; JAK, Janus-Kinase; BAFF, B cell activating factor belonging to TNF family; BCR, B-cell receptor; Lt-βR, Lymphotoxin-beta-receptor; CD40L, CD40 Ligand; ICOSL, Inducible co-stimulator ligand; CTLA4L, Cytotoxic T-Lymphocyte antigen 4 ligand. Created with www.BioRender.com, accessed on 13 October 2022.

As far as other monoclonal antibodies are concerned (Figure 2), abatacept (CTLA4–Ig), a T cell co-stimulatory signal modulator, showed promising results in an open-label study [113], yet these findings need further confirmation in larger trials. Anti-TNFα biologicals were not found to not improve symptoms and/or disease activity [114,115]. Similarly, disappointing results were observed with anti-IL-1 or anti-IL-6 monoclonals [116].

Targeting T-lymphocytes is another potential area of interest in pSS, as their inhibition/modulation would contribute to block macrophage activation and, thus, the chronicity of adaptive immune activation. In fact, drugs that block ICOS-ligand, a stimulator of T-lymphocyte pathways, are being studied with similar results to Baminercept (Lymphotoxin β receptor IgG fusion protein) but without favourable results [117]. CD40 ligand (CD40-L) inhibitors are also being studied to prevent T cell differentiation and activation [118].

The kinases and their signalling pathways could theoretically be targeted in pSS. These include the Bruton kinase Pi3K that is capable of reducing B cells overactivation, although, for the moment, existing drugs in clinical trials have not shown favourable results (Seletalisib, anti-Pi3K) [119].

Macrophages play an important role in signalling pathways. Although a safe depletion of macrophages has not been achieved, another approach would be to inhibit macrophage chemotactic proteins (MCPs) in order to prevent their destructive involvement in target tissues. These chemokines are: CCL2 (MCP-1)/CCR2, CX3CL1 (fractalkine)/CX3CR1, and CCL5 (RANTES)/CCR5.

In light of much evidence defining IFN as one of the main effectors in pSS pathogenesis, many classes of drugs interfering with its function and related pathways are now under investigation. Suppression of IFN-stimulated JAK-STAT signalling by oral JAK-inhibitors is currently considered in ongoing clinical trials (such as with tofacitinib, a dual JAK 1/2 inhibitor) [120]. In a pilot phase I/II proof-of-concept study, baricitinib proved effective in providing benefit in the majority of patients with high disease activity [121]. Lastly, Anifrolumab, a monoclonal antibody, which directly targets IFN-1 [122] and was recently approved for the treatment of SLE [123,124], is under investigation, and the results of current trials in pSS are eagerly awaited [125].

## 7. Conclusions

Sjögren’s syndrome is a very heterogeneous disease mostly led by dysfunction in lymphocyte function and activity, yet much evidence indicates that monocytes/macrophages are relevant in promoting inflammation, lymphocyte over-activation, and eventually structural damage. Monocytes and related signalling pathways (interferon signature) are emerging as new research targets whose relevance and precise characterisation have only recently started to be elucidated by transcriptome and epigenetic studies. The precise definition and role of monocyte-derived molecules involved in activation, inhibition, or regulation of the immune system within this intricate puzzle that is Sjögren’s disease are still far from completely understood. In this complex scenario, SS emerges as a pathological entity halfway between SLE and rheumatoid arthritis, with its own identity and few globally effective therapies so far.
